# Prognostic Value of Time-to-Positivity and Cycle Threshold (CT) Values in Predicting Tuberculosis Outcomes and Multidrug Resistance in Northern India: A Study Using Liquid Culture and Molecular Diagnostics

**DOI:** 10.7759/cureus.86087

**Published:** 2025-06-15

**Authors:** Raunak Bir, Urvashi B Singh, Hitender Gautam, Nishant Verma, Kiran Bala, Anant Mohan, Randeep Guleria

**Affiliations:** 1 Department of Microbiology, Employees' State Insurance Corporation (ESIC) Medical College and Hospital, Faridabad, IND; 2 Department of Microbiology, All India Institute of Medical Sciences, New Delhi, IND; 3 Department of Pulmonary, Critical Care and Sleep Medicine, All India Institute of Medical Sciences, New Delhi, IND; 4 Department of Pulmonary, Critical Care and Sleep Medicine, Medanta - The Medicity, Gurgaon, IND

**Keywords:** cycle threshold, multidrug resistance, prognosis, time to positivity, tuberculosis

## Abstract

Background: Relapse and default cases of pulmonary tuberculosis (PTB) present a significant challenge due to higher bacillary loads and the increased risk of developing multidrug-resistant tuberculosis (MDR-TB). Conventional diagnostic methods, such as smear microscopy, lack sensitivity and timeliness. This study evaluates the prognostic value of time to positivity (TTP) from liquid culture and cycle threshold (CT) values from GeneXpert *Mycobacterium tuberculosis*/rifampicin (Cepheid, Sunnyvale, CA) in predicting treatment outcomes.

Objectives: This study aimed to assess the utility of baseline mycobacteria growth indicator tube (MGIT) TTP and GeneXpert CTvalues as early biomarkers for prognosis and the risk of MDR-TB development in smear-positive relapse and default PTB patients.

Methods: This cross-sectional study with a prospective arm enrolled 72 adult PTB patients (relapse, default, or treatment failure). Sputum samples underwent smear microscopy, MGIT liquid culture, and GeneXpert testing. Baseline and three-month follow-up results were compared. TTP and CT values were correlated with microbiological outcomes.

Results: MGIT culture was positive in 52.8% (38/72) of patients (mean TTP: 26.5 days). In comparison, GeneXpert detected *M. tuberculosis* in 70.8% (51/72) (mean CT: 24.4). At follow-up, 7.8% (3/38) remained culture-positive (mean TTP: 29.7 days); one isolate was MDR. Patients with follow-up culture positivity had significantly lower baseline TTP (19.7 ± 3.4 vs. 25.8 ± 3.8 days). CT values trended lower among poor responders but did not reach statistical significance. Logistic regression suggested that a TTP threshold of ≤20.89 days might predict poor outcomes (model accuracy: 70%).

Conclusion: Baseline MGIT TTP demonstrates significant prognostic potential in identifying patients with PTB at risk of treatment failure or MDR development. In contrast, GeneXpert CT values, while useful diagnostically, were less predictive of outcomes. Incorporating TTP into routine clinical practice could enable earlier intervention and improve TB control strategies in high-burden settings.

## Introduction

Tuberculosis (TB), one of the major infectious causes of morbidity and mortality worldwide, remains a significant public health issue. The ongoing burden of TB is highlighted by the fact that, in 2023 alone, the World Health Organization (WHO) recorded about 10.8 million new cases and 1.25 million fatalities globally [1]. The TB epidemic continues to be severe in high-burden nations like India, which accounts for over a quarter of all cases worldwide [2], despite significant improvements in diagnosis, treatment, and control programs. The prevalence of TB in India is exacerbated by the development and transmission of drug-resistant strains, which pose significant challenges for TB management initiatives [3].

Patients with pulmonary tuberculosis (PTB) who are in the default or relapse group represent a distinct and vulnerable segment among those afflicted by the illness. These individuals have relapsed after completing their initial course of treatment, missed treatment, or experienced treatment failure [4]. They are often associated with higher bacillary loads, prolonged infectiousness, and an increased risk of carrying drug-resistant strains of *Mycobacterium tuberculosis*, particularly multidrug-resistant TB (MDR-TB) [5]. Due to the potential for ongoing transmission and the need for more complex and costly therapeutic plans [6], this population faces a greater risk of adverse clinical outcomes and poses a significant public health concern.

The primary method for tracking therapy response in TB is sputum smear microscopy and culture-based techniques. Because sputum smear microscopy is quick, easy, and inexpensive, it is frequently used. Nevertheless, it has certain drawbacks, such as its inability to distinguish between viable and nonviable bacilli and its reduced sensitivity in individuals with a low bacillary load or extrapulmonary illness [7]. Culture conversion, which is the shift from a positive to a negative culture result, remains the gold standard for determining a bacteriological response to treatment, but it takes several weeks for results to appear [8]. This delay may prolong unsuccessful treatment and increase the likelihood of developing drug resistance, which hampers prompt clinical judgment. For this reason, it is imperative to have early, quantitative, and sensitive biomarkers that can accurately forecast treatment outcomes, particularly in challenging situations [9].

Recent advancements in diagnostic methods have led to the development of promising instruments that may overcome these constraints. By continuously monitoring mycobacterial growth, the Mycobacteria Growth Indicator Tube (MGIT) 960 system enables automated and rapid liquid culture, providing the time to positivity (TTP) as an indirect measure of bacterial load and metabolic activity. Shorter TTP values indicate higher bacillary loads and more active infections [10]. Similarly, the GeneXpert *Mycobacterium tuberculosis* (MTB)/rifampicin (RIF) test, a nucleic acid amplification test approved by the WHO, can quickly identify rifampicin resistance and the DNA of *M. tuberculosis*. The cycle threshold (CT) values of the assay offer a semiquantitative estimate of bacterial DNA concentration, with lower CT values suggesting greater bacterial loads [11,12]. Although both TTP and CT values have been examined separately as indicators of illness severity and treatment response, their prognostic value, especially in smear-positive relapse and default PTB patients, remains under investigation [13].

This study aims to assess the potential of baseline MGIT TTP and GeneXpert CT values as early prognostic markers in smear-positive relapse and default PTB patients. By examining the relationship between these quantitative variables and treatment outcomes, we seek to identify reliable surrogate indicators that can enhance patient care. Early identification of poor responders would enable prompt adjustments to treatment regimens, reduce the likelihood of continued transmission, and support more effective TB control measures in high-burden countries like India.

## Materials and methods

This was a single-center, cross-sectional study with a prospective arm conducted at the Mycobacteriology Laboratory, Department of Microbiology, and the Department of Pulmonary Medicine and Sleep Disorders at the All India Institute of Medical Sciences, New Delhi, India, from September 2016 to October 2017 (IECPG:392/2016).

The microscopy grading results of the respiratory samples were compared with the CT values of GeneXpert MTB/RIF, and these results were simultaneously compared with liquid culture TTP by MGIT 960 from adult patients with PTB. The inclusion criteria targeted patients diagnosed with PTB who were experiencing relapse, treatment failure, or default, while patients with coexisting lung disorders were excluded.

During the study period, smear microscopy, GeneXpert MTB/RIF, and liquid culture were systematically performed on the respiratory samples. The samples were processed by decontamination with N-acetyl-L-cysteine-sodium hydroxide, followed by centrifugation and resuspension. Aliquots were then inoculated into MGIT tubes and prepared for smear microscopy. Clinical, radiological, and microbiological data were recorded from the requisition forms only.

Microscopy

All the respiratory samples received at the laboratory were subjected to smear microscopy for acid-fast bacilli by Ziehl-Neelsen (ZN) staining for semiquantitative assessment of bacterial load. ZN staining was performed by National TB Elimination Program guidelines, with bacterial load quantified based on the number of acid-fast bacilli observed per oil immersion field, classified as zero, sparse, 1+, 2+, or 3+ (2).

Liquid culture

The liquid culture process utilized the MGIT 960 system, which detects bacterial growth through oxygen-sensitive fluorescence while maintaining contamination control via Gram staining and cultivation on Mueller-Hinton agar. Positive MGIT cultures were identified for the *M. tuberculosis* complex using the BD MGIT™ TBc Identification Test (Becton, Dickinson and Company, Franklin Lakes, NJ), which detects the MPT64 antigen through a simple lateral flow assay; the presence of pink or red lines indicates a positive result. For drug susceptibility testing, rifampicin susceptibility was evaluated using the MGIT 960 system, with resistance defined by growth units exceeding a threshold of 100 in the presence of the drug. The procedure involved using inoculum from positive MGIT tubes and incubating until growth units reached 400, or resistance was determined if the growth units exceeded 100 [14].

Molecular diagnosis

The Xpert MTB/RIF (Cepheid Inc., Sunnyvale, CA) test utilizes heminested real-time polymerase chain reaction with molecular beacons to detect *M. tuberculosis* DNA and mutations associated with rifampicin resistance within the rpoB gene. Processed sputum was mixed with sample reagent, loaded into cartridges, and analyzed with CT values recorded to reflect bacterial load: high (Ct ≤ 16), medium (Ct 17-22), low (Ct 23-28), and very low (Ct 29-38). A Ct of 40 or higher was regarded as negative [13].

Follow-up

Follow-up assessments at three months involved repeat sputum testing, and subgroup analyses compared survivors to nonsurvivors and MDR to drug-susceptible cases.

Statistical analysis

Data were summarized and analyzed using the Statistical Package for the Social Sciences version 20.0 software (IBM Inc., Armonk, NY). Data were expressed as the mean, median, or number and percentage as appropriate. Normality was assessed using the Kolmogorov-Smirnov test. For the comparison of categorical data, the chi-square test or Fisher's exact test (with Yates’s correction if needed) was used. We derived receiver operating characteristic curves. A value of p less than 0.05 was considered statistically significant in the study.

## Results

Demographic characteristics

A total of 72 patients diagnosed with PTB, categorized into default, relapse, or retreatment groups, were enrolled in the study. The demographic profile revealed a male predominance, with 45 men (45/72; 62.5%), the highest proportion (12/72; 16.7%) in the 36-40 age group, followed equally by the 21-25, 26-30, and 31-35 age groups (each 8/72; 11.1%). The median age was 38 years, with a mean age of 40.3 ± 15.2 years.

Among the subcategories, relapse cases made up the majority at 53% (38/72), followed by defaulters at 29% (21) and treatment failures at 18% (13/72). Notably, individuals aged 26-30 were more prominent in the defaulter and treatment failure subcategories, whereas relapse cases were primarily found in the 36-40 age group. A male predominance was observed across all subcategories, particularly among relapse patients, with 25 out of 38 falling into this category.

Clinico-radiological presentation at baseline

At baseline, the most common clinical manifestations included cough, fever, weight loss, and dyspnea, while less frequent symptoms were loss of appetite, expectoration, and night sweats. The median durations of cough, fever, and expectoration were two, one, and one month, respectively. A history of Bacillus Calmette-Guérin vaccination was present in 43% (31/72) of patients. Substance abuse was noted in 41.7% (30/72) of patients. Comorbidities were present in 30 patients (41.7%), with hypertension being the most common (19/72; 26.4%), followed by diabetes mellitus (5/72; 6.9%). A positive family history of TB was documented in 16.7% (12/72) of participants. Chest X-rays performed on all patients showed radiological abnormalities in 49 patients (68%). Patchy infiltration was the most common finding (27/49; 55.1%) (Table 1).

**Table 1:** Clinical and radiological profile of study participants (baseline: n = 72, follow-up: n = 40) BCG: Bacillus Calmette-Guérin; TB: tuberculosis; STD: sexually transmitted infection

Parameter	Findings	Value, n (%)
Symptoms at baseline	Cough	72 (100%)
	Fever	52 (72.2%)
	Weight loss	57 (79.2%)
	Dyspnea	24 (33.3%)
	Expectoration	38 (52.8%)
	Loss of appetite	34 (47.2%)
	Night sweats	17 (23.6%)
	Hemoptysis	17 (23.6%)
BCG vaccination	History present	31 (43%)
	History absent	41 (57%)
Substance abuse	Any form	30 (41.7%)
	Smoking	18 (25%)
	Alcohol	12 (16.7%)
Comorbidities	Any comorbidity	30 (41.7%)
	Hypertension	19 (26.4%)
	Diabetes mellitus	5 (6.9%)
	Chronic hepatitis	3 (4.2%)
	HIV	2 (2.8%)
	Asthma	1 (1.4%)
Family history of TB	Positive	12 (16.7%)
History of STDs or transfusion	None reported	0 (0%)
Radiological findings	Any abnormality	49 (68%)
	Patchy infiltrates	27 (37.5%)
	Cavities	13 (18.1%)
	Consolidation	6 (8.3%)
	Combination	3 (4.2%)
Follow-up	Asymptomatic patients	31 (77.5%)
	Symptomatic patients	9 (22.5%)
Symptoms at follow-up	Cough	9 (12.5%)
	Expectoration	3 (4.2%)
	Fever	2 (2.8%)

Microbiological findings at baseline

At baseline, sputum smear microscopy using ZN staining was positive in 22 patients (22/72; 30.6%), while 50 patients (50/72; 69.4%) were smear-negative. MGIT demonstrated growth in 38 patients (38/72; 52.8%), with an average TTP of 26.5 days. No growth was observed in 34 patients after 42 days of incubation. The Gene Xpert MTB/RIF assay detected *M. tuberculosis *in 51 patients (51/72; 70.8%), with an average CT value of 24.4. Rifampicin resistance was not detected at baseline (Table 2).

**Table 2:** Microbiological profile of study participants (baseline: n = 72, follow-up: n = 40) ZN: Ziehl-Neelsen; MGIT: mycobacteria growth indicator tube; TTP: time to positivity

Parameter	Findings	n (%)/values
ZN staining (baseline)	Positive	22 (30.6%)
	Negative	50 (69.4%)
	Scanty	1 (1.4%)
	1+ grading	8 (11.1%)
	2+ grading	8 (11.1%)
	3+ grading	5 (6.9%)
MGIT liquid culture (baseline)	Positive	38 (52.8%)
	Average TTP	26.5 days
	TTP 15-20 days	4 (5.6%)
	TTP 21-25 days	14 (19.4%)
	TTP 26-30 days	18 (25%)
	TTP 31-35 days	2 (2.8%)
	No growth (42 days)	34 (47.2%)
GeneXpert (baseline)	Positive	51 (70.8%)
	Average C_T_ value	24.4
	Very-low-grade C_T_	11 (15.3%)
	Low-grade C_T_	23 (31.9%)
	Medium-grade C_T_	13 (18.1%)
	High-grade C_T_	4 (5.6%)
	Rifampicin resistance	0
ZN staining (follow-up)	Positive	1 (2.5%)
MGIT liquid culture (follow-up)	Positive	3 (7.5%)
	Average TTP	29.7 days
	Rifampicin resistance	1 (2.5%)
Follow-up MGIT TTP	Positive patients	19.7 ± 3.4 days
	Negative patients	25.8 ± 3.8 days (p = 0.017)
Follow-up GeneXpert C_T_	Positive patients	18.7 ± 5.5
	Negative patients	24.7 ± 3.8 (p = 0.08)
Death reported	One patient (smear 3+, TTP 18 days, C_T_ 16)	

Microbiological follow-up findings

Of the initial 72 patients, 40 (55.7%) were followed up three months after the start of treatment. Thirty-two were lost to follow-up, including three deaths. At follow-up, nine patients (9/40; 22.5%) reported symptoms, all of whom experienced a cough (Table 2).

ZN staining was positive in one patient (1/40; 2.5%), while liquid culture was positive in three patients (3/40; 7.5%), with an average TTP of 29.7 days. One isolate exhibited rifampicin resistance and was classified as MDR.

One patient died within two months of initiating treatment, presenting with a high baseline smear grade (3+), a short TTP of 18 days, and a low CT value of 16, indicating a heavy bacterial burden.

Microbiological correlations and statistical analysis

**Figure 1:**
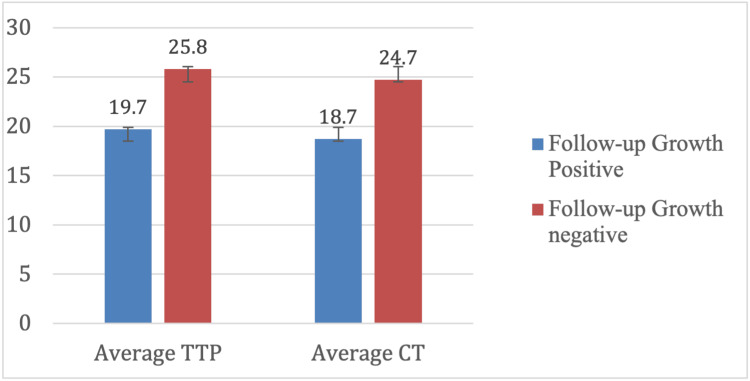
Comparison of average TTP and average CT value TTP: time to positivity; CT: cycle threshold

Baseline mean TTP among the three patients with positive MGIT growth at follow-up was significantly lower (19.7 ± 3.4 days) compared to the 17 patients who remained culture negative (25.8 ± 3.8 days) (p = 0.017). The average CT value was lower in MGIT-positive patients (18.7 ± 5.5) compared to MGIT-negative patients (24.7 ± 3.8), though this difference was not statistically significant (p = 0.08) (Figure 1).

The association between TTP and treatment outcome was statistically significant, as demonstrated by a chi-square test (p = 0.019). To evaluate the prognostic utility of TTP, binary logistic regression was performed, yielding a significant model (p = 0.036) with a predictive accuracy of 70%. The model's Nagelkerke R² value was 0.291, indicating that TTP accounted for approximately 29% of the variability in treatment outcomes. Although the regression coefficient for TTP approached significance (p = 0.075), it did not meet the conventional threshold, with the model intercept and slope calculated as 6.004 and -0.299, respectively, suggesting a TTP threshold of 20.89 days. In contrast, no significant correlation was found between CT values and outcomes. Additionally, a multinomial regression analysis based on patient category revealed no significant influence of covariates on the primary outcome, further supporting the potential of TTP as an independent prognostic marker.

## Discussion

This study examined the demographic, clinical, radiological, and microbiological profiles of patients with default and relapse TB who underwent retreatment, emphasizing the prognostic value of microbiological markers, including TTP in liquid culture and GeneXpert CT values. The observed male predominance (62.5%) and concentration in the 36-40 years age group align with global TB epidemiology, which often shows higher incidence among middle-aged men, potentially due to greater exposure to risk factors such as smoking and substance abuse [15,16]. Furthermore, the presence of comorbidities, notably hypertension and diabetes mellitus, corresponds with existing literature that identifies these conditions as significant contributors to TB susceptibility and treatment complexity [17,18].

Baseline ZN smear positivity was 30.6%, whereas MGIT liquid culture positivity was higher at 52.8%, confirming the superior sensitivity of culture-based methods over smear microscopy [19]. GeneXpert MTB/RIF detected 70.8% positivity, reflecting its ability to identify bacterial DNA regardless of viability [20]. No rifampicin resistance was identified at baseline, suggesting either a low prevalence in this cohort or early-stage drug resistance.

During follow-up, smear and culture positivity decreased to 2.5% and 7.5%, respectively, indicating effective bacterial clearance consistent with other retreatment studies [21]. The identification of one rifampicin-resistant isolate during follow-up underscores the persistent challenge of emerging drug resistance [22].

A significantly shorter baseline TTP in patients with poor outcomes supports the use of TTP as a surrogate marker for bacillary load and disease severity. This aligns with prior studies demonstrating the predictive value of TTP for treatment response and risk of relapse [23,24]. Although the logistic regression did not reach conventional significance thresholds (p = 0.075), the trend suggests that TTP merits further investigation as a potential prognostic tool.

Conversely, GeneXpert CT values showed no significant correlation with treatment outcomes, likely because CT values measure both live and dead bacterial DNA, which limits their prognostic accuracy [12,20]. This highlights the complementary roles of GeneXpert for diagnosis and TTP for monitoring treatment response.

The study was limited by the loss to follow-up cases, which constrained the ability to generalize findings and fully assess long-term outcomes. Nonetheless, the findings support incorporating TTP measurement into the routine clinical management of retreatment TB patients. Early identification of patients with shorter TTP could enable intensified follow-up for treatment failure and the development of multidrug resistance, potentially improving outcomes amid rising concerns about drug resistance.

## Conclusions

Default and relapse PTB patients exhibit diverse clinical and microbiological profiles, with TTP emerging as a promising prognostic biomarker. While GeneXpert remains essential for rapid diagnosis and resistance detection, TTP may provide better predictions of treatment success and help identify high-risk patients. Larger prospective studies, including patients with extrapulmonary TB, are needed to validate these results and refine retreatment strategies.
